# Impairing hydrolase transport machinery prevents human melanoma metastasis

**DOI:** 10.1038/s42003-024-06261-y

**Published:** 2024-05-15

**Authors:** Alice Nordlinger, Justine Del Rio, Shivang Parikh, Laetitia Thomas, Roma Parikh, Hananya Vaknine, Ronen Brenner, Francesco Baschieri, Aude Robert, Mehdi Khaled

**Affiliations:** 1 INSERM 1279, Tumor Cell Dynamics, Gustave Roussy, Université Paris-Saclay, Villejuif, France; 2 The Ragon Institute of MGH, MIT, and Harvard University, Cambridge, MA USA; 3 Institute of Pathology, E. Wolfson Medical Center, Holon, Israel; 4 Present Address: Institute of Pathophysiology, Innsbruck, Austria

**Keywords:** Metastasis, Cancer

## Abstract

Metastases are the major cause of cancer-related death, yet, molecular weaknesses that could be exploited to prevent tumor cells spreading are poorly known. Here, we found that perturbing hydrolase transport to lysosomes by blocking either the expression of *IGF2R*, the main receptor responsible for their trafficking, or GNPT, a transferase involved in the addition of the specific tag recognized by IGF2R, reduces melanoma invasiveness potential. Mechanistically, we demonstrate that the perturbation of this traffic, leads to a compensatory lysosome neo-biogenesis devoided of degradative enzymes. This regulatory loop relies on the stimulation of *TFEB* transcription factor expression. Interestingly, the inhibition of this transcription factor playing a key role of lysosome production, restores melanomas’ invasive potential in the absence of hydrolase transport. These data implicate that targeting hydrolase transport in melanoma could serve to develop new therapies aiming to prevent metastasis by triggering a physiological response stimulating *TFEB* expression in melanoma.

## Abstract (web summary)

An in vitro and in vivo study using human melanoma cell lines suggests that perturbing lysosomal function stimulates lysosome biogenesis and decreases invasiveness potential.

## Introduction

Although immunotherapies have proven their efficacy by inducing long-term response for half of eligible metastatic melanoma patients, yet, the remaining patients encounter failure due to primary or acquired resistance. The deadliness of melanoma is directly linked to his high metastatic potential. Therefore, there is an urgent need to find treatments that could prevent or delay metastasis. The mechanisms used by melanoma to escape the primary tumor and initiate invasion are very complex. This transition has been described to be mediated by the microenvironment and to rely on transcriptional plasticity. This plasticity is mainly based on the expression of *MITF* transcription factor as it has been shown that modulating its expression can either promote proliferation or invasion^1^. Therefore, single molecular targets are a viable strategy to profoundly affect melanoma transcriptome in order to prevent metastasis. As MITF has a dual role in tumor progression, it cannot serve as a therapeutic target. We therefore evaluated the opportunity of targeting genes within its downstream network. Among those targets, the expression of *IGF2R* has been shown to be necessary for melanoma invasion by an unknown mechanism^2^. Since this receptor is modular and binds different ligands, we reasoned that it could be feasible to block only the function involved in melanoma metastasis without affecting other cellular function regulated by IGF2R. The insulin-like-growth factor 2 receptor (IGF2R) is a type 1 transmembrane glycoprotein involved in multiple cellular functions. Its large extra-cytoplasmic tail contains 15 repeat domains to bind different ligands depending on its subcellular localization^3^. When it is addressed to the plasma membrane, IGF2R internalizes excessive extracellular IGF2^4,5^ but can also activate latent TGFβ through Urokinase receptor and plasmin fixation^6,7^. However, its major function occurs in the trans-Golgi network and consists in transporting soluble lysosomal hydrolases to lysosomes, where they mediate macromolecule degradation. This transport depends on the recognition of a mannose-6-phosphate tag (m6p) added by a two-step reaction. First, an *N*-acetylglucosamine-1-phosphate is added to the high mannose oligosaccharide chains of the newly synthesized hydrolases by the hexameric enzyme UDP-*N*-acetylglucosamine:lysosomal enzyme N-acetylglucosamine-1-phosphotransferase (GNPT)^8^. GNPT is formed by two alpha (GNPTA) and beta subunits (GNPTB), both coded by *GNPTAB* gene and two gamma subunits (GNPTG) coded by *GNPTG* gene^9^. Then, the uncovering enzyme N-acetylglucosamine-1-phosphodiester α-N-acetylglucosaminidase (NAGPA) removes the N-acetylglucosamine to reveal the m6p recognized by mannose 6-phosphate receptors (MPRs)^10^.

Among IGF2R ligands, IGF2^11^, TGFβ^12^, uPAR, and plasminogen^13^ are known to be important in invasion, but the functional consequences of loss of *IGF2R* on cancer cell dissemination are unclear. Circulating and tissue levels of IGF2 are regulated by IGF2R^14^; therefore,  downregulation of *IGF2R,* increases the level of IGF2 resulting in a stimulation of IGF1R signaling, which should promote invasion. The binding of TGFβ1 to IGF2R leads to its activation^6^. Consequently, the  downregulation of *IGF2R* could lead to a decrease in the level of activated TGFβ1, which should inhibit invasion. In the case of uPAR and plasminogen, the downregulation of *IGF2R* will lead to a decrease of plasminogen cleavage and activation, which should also inhibit invasion. Given this, it seemed likely that either TGFβ1 or plasminogen were responsible for pro-invasive features of IGF2R. Interestingly TGFβ1 and plasminogen have been shown to be upregulated during cancer cell invasion^12,13^. Due to this reasoning, we directly tested whether TGFβ and plasminogen were involved in melanoma invasion and we observed that neither of the activated factors alone were able to restore melanoma invasion upon *IGF2R* knockdown. Next, we investigated if impairing the role of IGF2R in hydrolase transport could affect melanoma invasiveness potential. IGF2R recognizes a mannose-6- phosphate tag added to hydrolases by GlcNAc-phosphotransferase during their maturation in the Golgi. Remarkably, we found that lysosomal hydrolase transport is a key step in cancer invasion, a property that was never attributed to the lysosome function. Indeed, *GNPTAB* depletion by siRNAs, phenocopied the depletion of *IGF2R* and reduced melanoma invasiveness potential. Loss of *GNPTAB* or *IGF2R* expression also induced a significant increase in lysosome number. Interestingly, *GNPTAB* or *IGF2R* downregulation led to an increase of the transcription factor EB (TFEB), a well-known regulator of lysosomal function^15^, demonstrating a regulatory loop between lysosome function and *TFEB* expression. Taken together, our results revealed the association of lysosome function to melanoma metastasis via the *GNPTAB*, *IGF2R*, and *TFEB* axis. Our work proposes a strategy to take benefits of melanoma physiological reaction to lysosomal stress in order to prevent melanoma invasion.

## Results

### Melanoma invasion regulation by IGF2R is not mediated by TGFβ nor plasmin

**Fig. 1:**
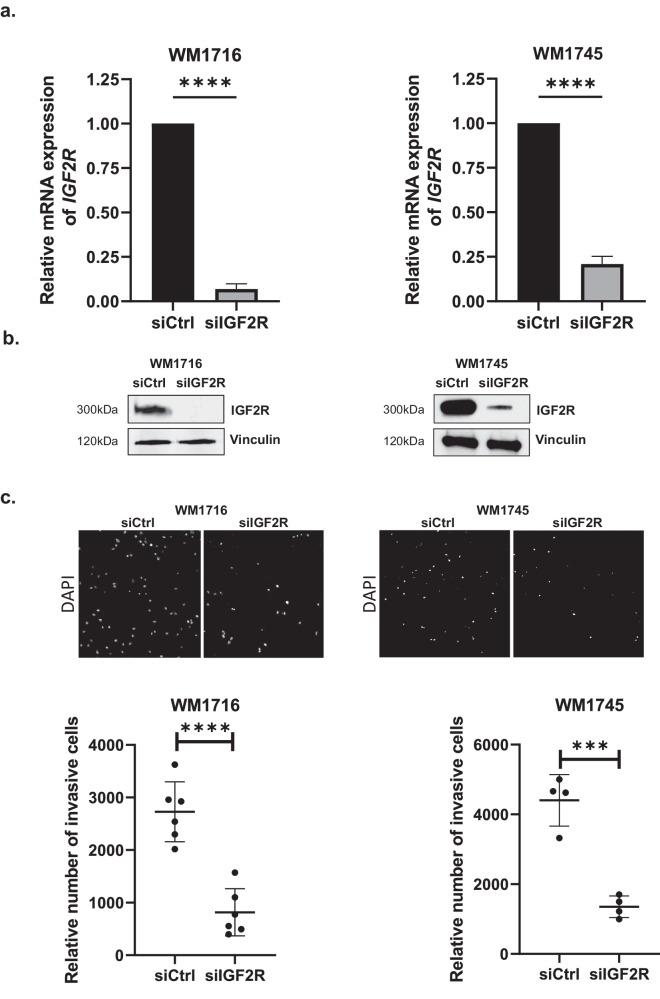
Inhibition of *IGF2R* expression prevents melanoma invasion. **a** *IGF2R* mRNA expression level in WM1716 (*n* = 10 independent experiments) and WM1745 (*n* = 5 independent experiments) siCtrl or siIGF2R transfected cells. Data are normalized to actin and relative to transfection with siCtrl. **b** Protein extracts from WM1716 and WM1745 cells transfected with *IGF2R* siRNA were analyzed by western blot using IGF2R antibody. Vinculin served as a loading control. **c** Relative number of invasive melanoma cells WM1716 and WM1745, measured by Matrigel coated transwell assays, after transfection with siCtrl or siIGF2R (*n* = 6 and *n* = 4 independent experiments respectively). Upper panels are showing representative field of Boyden chambers’ membrane. Lower panels are graphical representation of the data. Unpaired t-test were used for statistical analysis. Error bars represent ± SD, ***p < 0.001, ****p < 0.0001.

**Fig. 2:**
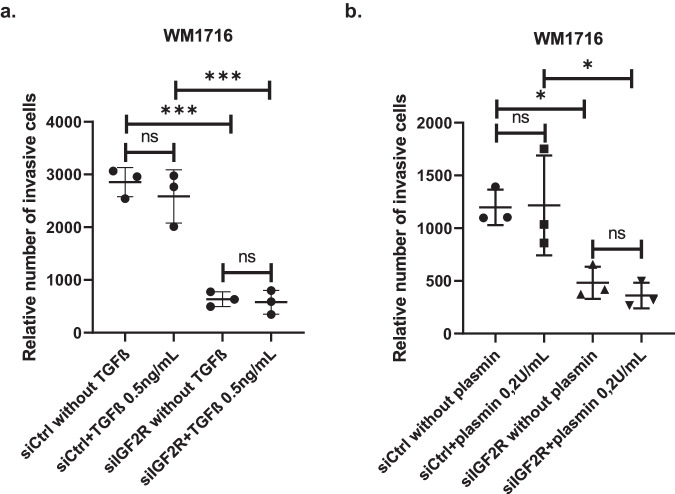
Supplementation of WM1716-siIGF2R depleted cells with active TGFβ or active plasmin cannot rescue melanoma invasiveness potential. **a** Relative number of WM1716 invasive cells after transfection with siCtrl or siIGF2R and treatment with recombinant active TGFβ at 0.1 or 0.5 ng/mL for 16 h (*n* = 3 independent experiments). **b** Relative number of WM1716 invasive cells after transfection with siCtrl or siIGF2R and treatment with recombinant plasmin at 0.2 or 0.5 U/mL for 16 h (*n* = 3 independent experiments). One way ANOVA tests were used for statistical analysis. Error bars represent ± SD, *p < 0.05, **p < 0.01, *** *p* < 0.001,**** *p* < 0.0001.

We previously demonstrated that *MITF* expression is inversely correlated with invasiveness potential of cultured melanoma cell lines^2^. In an effort to decipher how low *MITF* expression drives this phenotype, we identified IGF2 receptor as negatively correlated with *MITF* expression and playing a key role in invasion^2^. Indeed, inhibition of *IGF2R* expression using siRNA prevented melanoma invasion in low *MITF* expressing and highly invasive short-term culture melanoma cell lines WM1716 and WM1745 (Fig. 1a–c), which is in accordance with our previous study ^2^. However, the mechanism by which this receptor interferes with invasion was not known. To identify its mode of action, we first tested whether IGF2R ligands, namely TGFβ and plasminogen, modulate its effect on invasion, since both are known to be involved in metastasis^16^. We performed invasion assays on WM1716 human melanoma cells depleted for *IGF2R* and supplemented the media with either active TGFβ or the activated form of plasminogen, plasmin. Our data indicated that although the addition of active TGFβ induced its signaling, as seen by the induction of SMAD2 phosphorylation (Supplementary Fig. 1a), this cytokine could not rescue the invasive phenotype in the absence of IGF2R (Fig. 2a). Similarly, although plasmin demonstrates its functionality by inducing cleavage of MMP2 (Supplementary Fig. 1b), its addition could not rescue the invasive phenotype under *IGF2R* depletion (Fig. 2b). Taken together, our data demonstrate that activation of TGFβ or plasminogen by IGF2R is not necessary for melanoma invasion.

### The hydrolase transport machinery is required for invasiveness potential of melanoma

**Fig. 3:**
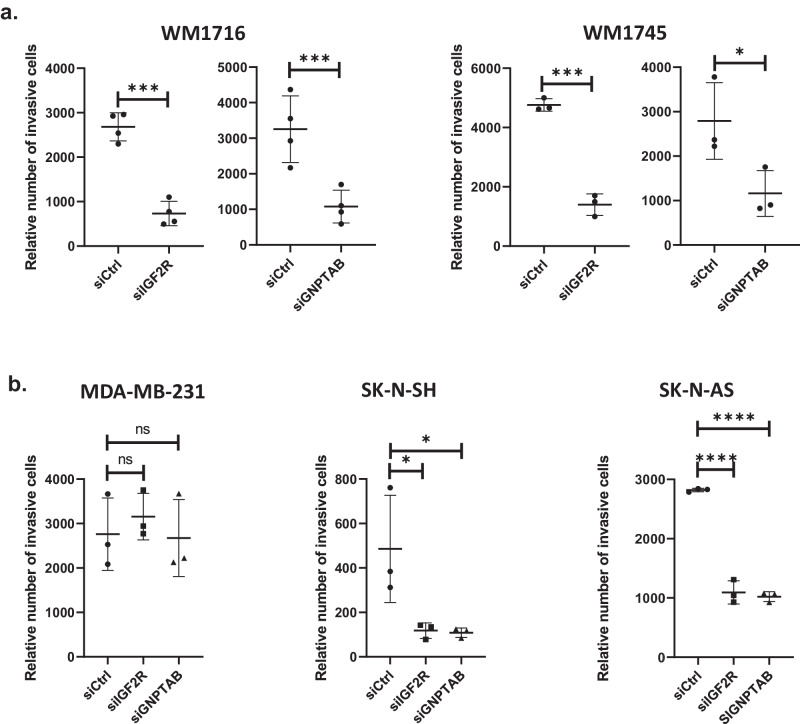
The hydrolases transport machinery depletion decreases invasiveness potential of melanoma cells. **a** Relative number of highly invasive melanoma cells WM1716 and WM1745 transfected with indicated siRNA (*n* = 5 independent experiments). **b** Relative number of invasive breast carcinoma MDA-MB-231 and neuroblastoma SK-N-SH and SK-N-AS cancer cell lines, transfected with indicated siRNA (*n* = 3 independent experiments). One way ANOVA tests were used for statistical analysis. Error bars represent ± SD, *p < 0.05, ***p < 0.001, ****p < 0.0001.

The major function of IGF2R is to transport newly formed hydrolases to lysosomes by recognizing the mannose-6-phosphate tag added during their maturation in the Golgi^3^. To determine if hydrolase transport could affect melanoma invasiveness potential, we tested the effect of inhibiting the mannose-6-phosphate tagging of hydrolases. We observed that inhibiting the expression of the alpha and beta subunit of GNPT, in WM1716 as well as WM1745, phenocopied the effect of *IGF2R* inhibition on melanoma invasion (Fig. 3a). Unlike our findings in melanoma, IGF2R has been described to be a tumor suppressor in gastrointestinal and breast cancer^17,18^, we therefore investigated the consequences of blocking the hydrolase transport machinery on non-melanoma cancer types. Inhibition of *IGF2R* or *GNPTAB* had no effect on ductal breast carcinoma cancer cell line MDA-MB-231 invasion, however it drastically inhibited the invasiveness potential of neuroblastoma cell lines SK-N-SH and SK-N-AS, probably since their share the same developmental origin as melanoma, namely neural crest (Fig. 3b and Supplementary Fig. 2a). Altogether those results indicate that inhibiting *IGF2R* expression reduces melanoma invasive potential by impairing hydrolase transport to the lysosomes.

### Loss of hydrolase transport machinery leads to increased lysosome number and *TFEB* expression

**Fig. 4:**
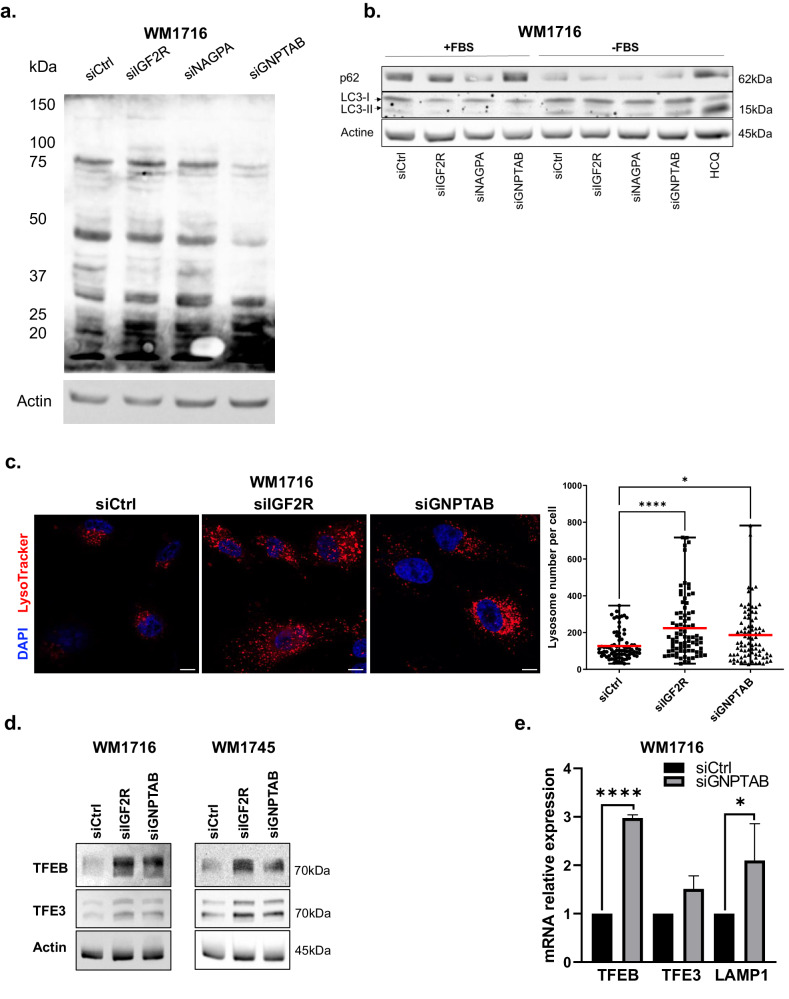
*IGF2R* and *GNPTAB* inhibition increase lysosome number and stimulate the expression of *TFEB* and *TFE3.* **a** Protein extracts from WM1716 cells transfected with indicated siRNA were analyzed by far western blot using biotinylated bovine IGF2R as a bait for mannose-6-phosphate proteins. Actin served as a loading control. **b** Protein extracts from WM1716 cells treated with indicated siRNA or lysosomotrope inhibitors hydroxychloroquine (HCQ, 50μM for 16 h) were analyzed for presence of autophagic markers p62 and LC3-II conjugated form in normal culture conditions (+FBS) or after autophagy induction in a nutrient-depleted medium (-FBS). Actin served as a loading control. **c** Immunofluorescence confocal microscopy images of WM1716 cells transfected with indicated siRNA and stained with Red LysoTracker (red) and DAPI (blue). The number of lysosomes per cell was quantified using Volocity software. Central bars indicate the average number of lysosomes per cell (right panel). Kruskal and Wallis tests were used for statistical analysis. Error bars represent ± SD, *p < 0.05, ****p < 0.0001. Scale bar = 10 μm. **d** Protein extracts from WM1745 and WM1716 cells transfected with indicated siRNA were analyzed by western blot using antibodies against TFEB, TFE3 and actin. **e** *TFEB*, *TFE3* and *LAMP1* mRNA expression level in WM1716 siRNA transfected cells. Data are normalized to actin and relative to transfection with siCtrl (*n* = 3–6 independent experiments). An unpaired t-test was used for statistical analysis. Error bars represent ± SD, ****p < 0.0001, *p < 0.05.

In an effort to understand the molecular basis of the involvement of hydrolase transport in melanoma invasion, we investigated the consequences of blocking this pathway on cell physiology. Using biotinylated soluble IGF2R as a bait for far western experiments, we first validated that inhibition of *GNPTAB* expression led to significantly less mannose-6-phosphate tagged protein. As expected, the depletion of *IGF2R* did not affect hydrolase tagging (Fig. 4a and Supplementary Fig. 2b). As a second control, we depleted cells from *NAGPA*, the enzyme acting downstream of GNPT and observed no differences in m6p tagged protein expression. These findings are in accordance with the literature as the uncovering of the m6p has been shown to not be required for hydrolase recognition by IGF2R^19^.

Targeting hydrolase transport to lysosomes should in theory affect the function of these organelles. Lysosome function is key in the final step of the autophagy process^20^. Since it was not clear to us how a lysosomal loss of function could be responsible for decreasing melanoma invasiveness potential, we tested in our experimental conditions if autophagy was affected. In this aim, we monitored the expression of LC3-I and II as well as p62 in cells grown in depleted or complete media. We observed in WM1716 melanoma cell line, that neither, n*IGF2R* or *GNPTAB* inhibition, interfered with autophagy markers, although in those conditions invasiveness potential was reduced. As expected, serum depletion induced a moderate autophagy and hydroxychloroquine, an agent blocking autophagy flux, used as positive control, induced  LC3-II conversion (Fig. 4b). Next, we tested the consequences of short-term inhibition of hydrolase transport machinery on lysosome number using Lysotracker and observed a strong staining increase (Fig. 4c and Supplementary Fig. 3a) in both WM1716 and WM1745 cell lines. Since lysosome biogenesis is mainly controlled by TFEB transcription factor^15^, we monitored *TFEB* expression under *IGF2R* or *GNPTAB* inhibition and observed that both conditions, stimulated its expression at mRNA and protein level (Fig. 4d, e). We also observed that TFE3, a transcription factor from the same family ^21^, was slightly increased at mRNA and protein levels in both WM1716 and WM1745 short-term culture melanoma cell lines (Fig. 4d, e and Supplementary Fig. 3b). The role of hydrolase transport in increasing lysosome production was further confirmed in WM1716 cells by the stimulation of *LAMP1* expression, a lysosome marker, under *GNPTAB* inhibition (Fig. 4e). In other cellular systems, TFEB activity has been described to be regulated by its translocation from the cytoplasm to the nucleus instead of its expression^22^. We therefore investigated its localization by subcellular fractionation and observed that in WM1716 melanoma cell line, TFEB was present in both nucleus and cytoplasm (Supplementary Fig. 4a). Since under nutrient depletion TFEB translocation to the nucleus is regulated by mTOR^23,24^, we tested if *GNPTAB* inhibition can affect this pathway by monitoring the phosphorylation of one of its target, 4E-BP1 and did not observe any effect (Supplementary Fig. 4b). These data revealed a regulatory loop between lysosome function and *TFEB* expression independently of canonical mTOR pathway. Further, our observations suggested that perturbing hydrolase transport in melanoma stimulates lysosome biogenesis as a consequence of *TFEB* expression upregulation.

### Blocking hydrolase transport stimulates lysosome biogenesis and reduces melanoma invasiveness potential though the upregulation of *TFEB* expression

**Fig. 5:**
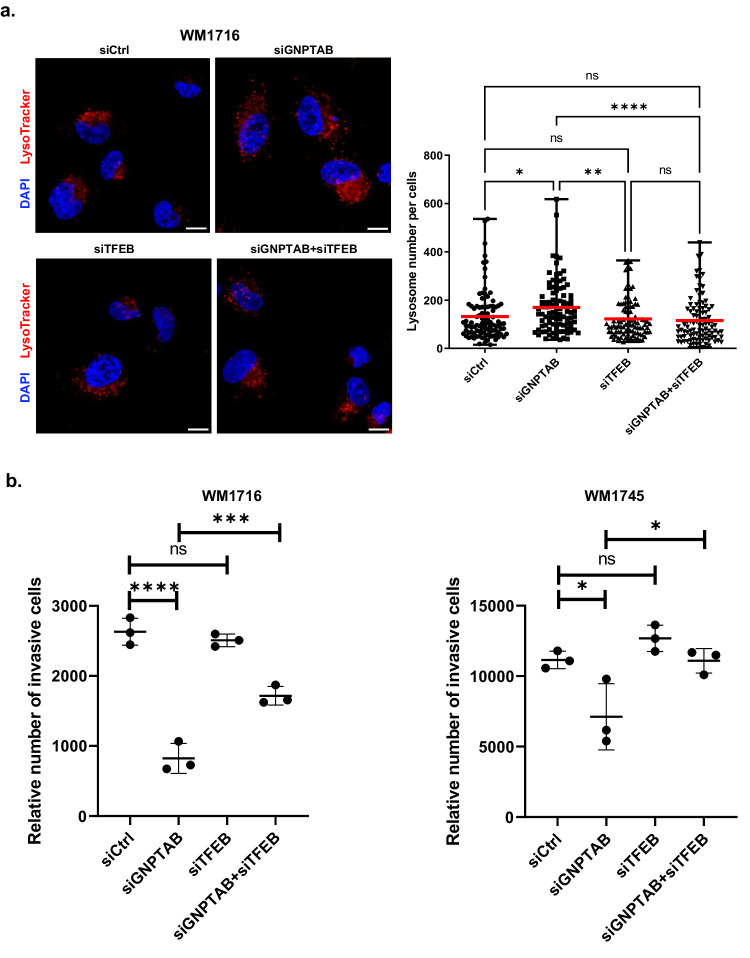
Modulation of *TFEB* expression regulates lysosome biogenesis and invasive potential of melanoma cells. **a** WM1716 cells were transfected with indicated siRNA and stained with Red LysoTracker and DAPI (left panel). The number of lysosomes per cell was quantified using Volocity software. Central bars indicate the average number of lysosomes per cell (right panel). Kruskal and Wallis tests were used for statistical analysis. Error bars represent ± SD, *p < 0.05, **p < 0.01, ****p < 0.0001. Scale bar = 10 μm. **b** Relative number of invasive melanoma cells WM1716 and WM1745 after transfection with indicated siRNA (*n* = 3 independent experiments). Kruskal and Wallis tests were used for statistical analysis. Error bars represent ±SD, * *p* < 0.05, *** *p* < 0.001, ****p < 0.0001.

To confirm that *TFEB* upregulation was responsible for the increased biogenesis of lysosomes, we transfected WM1716 cell line with either *GNPTAB* or *IGF2R* siRNA alone or together with siRNA specific for *TFEB* and then stained them with Red Lysotracker to quantify the number of lysosomes per cell (Fig. 5a and Supplementary Fig. 5). As expected, increased lysosome biogenesis following hydrolase transport perturbation by blocking either *IGF2*R or *GNPTAB* is TFEB dependent (respectively Supplementary Fig. 5 and Fig. 5a). Since TFEB and MITF are part of the same transcription factor family, and since they share some target genes^25^, we wondered if *TFEB* upregulation was responsible of the reduction of the invasiveness potential observed when hydrolase transport was perturbed. In this aim, we performed an invasion assay using WM1716 and WM1745 cell lines, transfected with siRNA specific for *GNPTAB* alone or together with siRNA specific for *TFEB*. Our data demonstrated that inhibition of *TFEB* expression rescued the invasiveness potential of both melanoma cell lines (Fig. 5b) depleted for *GNPTAB*. These data indicate that the upregulation of this transcription factor can block invasion, similarly to MITF. This was further confirmed by ectopically expressing TFEB. Indeed we observed that WM1716 cell line ectopically expressing this transcription factor exhibit a lower invasive potential than control cells (Supplementary Fig. 6a–c).

### Impairing hydrolase transport reduces melanoma metastasis

**Fig. 6:**
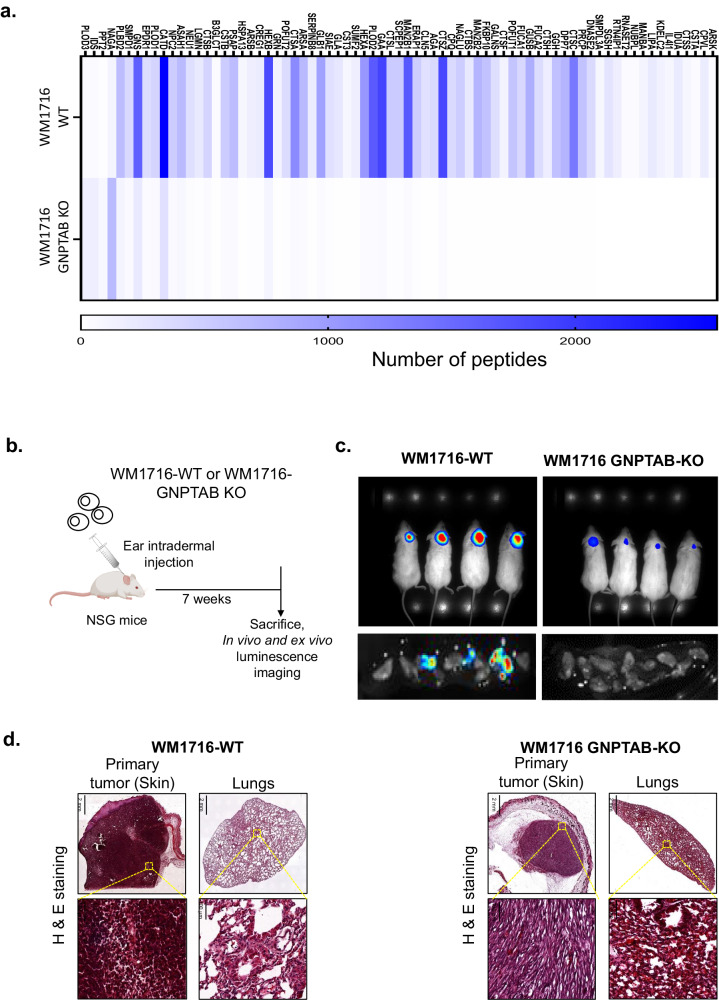
*GNPTAB* inhibition impairs tumor growth and metastasis. **a** Heat map representing a mass spectrometry analysis of the abundance of peptides harboring a mannose-6-phosphate tag in WM1716 WT or GNPTAB KO cell lines. The graphic represents the sum of peptides from four independent cultures and purification on immobilized IGF2R columns. The list of proteins was restricted to those associated with an adjusted *p*-value < 10^−5^. **b** Experimental design of melanoma cell lines engraftment. Mouse image was created with BioRender.com (**c**) WM1716 WT or GNPTAB KO cell lines both expressing luciferase were engrafted into immunodeficient mice. Metastasis and tumor development were monitored 7 weeks later using live imaging. Image of bioluminescence obtained in entire body or in lungs is shown for each melanoma cell line injected. **d** H&E staining of sections of the tumor at injection site or lungs taken from mice injected with WM1716 WT or GNPTAB KO cell lines.

Next, we examined our hypothesis of lysosome role in melanoma development in a physiologically relevant in vivo melanoma model. First, we generated melanoma cell lines deleted for *GNPTAB* using CRISPR/Cas9. After verifying efficient *GNPTAB* knock out (Supplementary Fig. 7a) in WM1716 cell line, we measured proliferation rate and observed that *GNPTAB* knock-out cells were slightly less proliferative than WM1716-WT (Supplementary Fig. 7b). Indeed, cell density of *GNPTAB* KO-WM1716 cell line four days after seeding was comparable to cell density of WT cell line at third day. We completed the validation of the generated cell line by verifying an accumulation of LC3-II which indicated its inability to complete autophagy ^26^, and therefore inability to produce functional lysosomes as a consequence of long-term inhibition (Supplementary Fig. 7c). This result was confirmed by analyzing Cathepsin B activity (Supplementary Fig. 7d) and by quantifying by mass spectrometry the abundance of protein tagged with m6p (Fig. 6a). Further, total depletion of *GNPTAB* reduced the invasiveness potential of the cell line in vitro (Supplementary Fig. 7e) and stimulated *TFEB* and in much less extent *TFE3* expression (Supplementary Fig. 7f, g). Finally, we observed that the invasive potential of *GNPTAB* KO-WM1716 melanoma cell line can be stimulated by inhibition of *TFEB* using specific siRNA (Supplementary Fig. 7h), recapitulating our observations made solely with transient gene interference.

Next, we engrafted WT-WM1716 and *GNPTAB* KO-WM1716, previously transduced with a lentivirus expressing luciferase into immuno-deficient NSG mice, experimental design is shown in Fig. 6b. As observed in vitro, the invalidation of *GNPTAB* led to a slightly reduced tumor growth at the injection site (Fig. 6c, upper panel). To investigate the ability of melanoma cells to successfully colonize distant organs, mice were sacrificed 7 weeks post primary tumor injection, and the lung were harvested to be incubated with luciferase substrate. We found that *GNPTAB* knockout cells exerted a significant decrease in melanoma metastatic potential (Fig. 6c, lower panel). The staining with H&E of the primary tumor and of lung sections confirms that inhibition of *GNPTAB* protected the lungs from micro-metastasis (Fig. 6d). Interestingly, Ki67 staining of the engrafted tumor showed no significant proliferation differences between the two cell lines (Supplementary Fig. 7i). Altogether, those results confirmed that perturbing hydrolase transport and thus increasing lysosome production, prevented melanoma metastasis.

## Discussion

The role of IGF2R in melanoma is rather unexpected, since this gene is generally considered as a tumor suppressor because loss of heterozygosity, SNPs, and somatic mutations of *IGF2R* are associated with several cancers^17,18,27^. Since IGF2R is a tumor suppressor in some tumor types, we wanted to determine whether our observations were restricted to melanoma. We therefore tested the effect of inhibition of expression of *IGF2R* and *GNPTAB* in several solid tumor cell lines. This treatment did not significantly affect the invasiveness of breast carcinomas which is  in agreement with a previous study^28^, indicating that our findings are not due to a technical artifact.

Although melanocytes are found in an epithelium, they do not derive from epithelial cells, rather, they originate from the neural crest^29^. Therefore we tested the role of hydrolase trafficking in neuroblastoma invasiveness because these tumor cells share an embryological origin with melanocytes. Inhibition of expression of either *IGF2R* or *GNPTAB* impaired invasiveness of neuroblastoma cell lines SK-N-SH and SK-N-AS. Thus, hydrolase trafficking is crucial for a subset of cancer types. This claimed is supported by a study demonstrating an increased expression of *IGF2R* in adrenocortical carcinomas compared with begnin adrenocortical adenomas and both originate from the neural crest^30^.

IGF2R is not the only intracellular receptor involved in hydrolase trafficking. This function is shared with the calcium-dependent mannose-6-phosphate receptor (CD-M6PR), which also recognizes protein tagged with an m6p motif. It is not clear whether these two receptors recognize the same tagged proteins, although there are some data to suggest that this is the case^31^. Unlike IGF2R, CD-M6PR only functions to target proteins to lysosomes. Because *IGF2R* knockout in mice causes neonatal lethality^4^, mainly because of its role in the regulation of IGF2-mediated signaling^5^, it is not possible to use these mice to determine whether CD-M6PR can fully compensate for the role of IGF2R. *CD-M6PR*-null mice do not exhibit any obvious phenotype but it was reported that some hydrolases are misrouted in those mice, indicating that IGF2R cannot fully compensate for the loss of CD-M6PR^32,33^. We therefore postulated that if the routing of hydrolases to lysosomes is indeed important for melanoma progression, the inhibition of *CD-M6PR* expression will affect melanoma invasion. As expected, down regulation of this receptor, decreased melanoma invasiveness potential confirming that impairing hydrolase transport specifically, affects melanoma invasion.

Since the misrouting of hydrolases has been described to induce their secretion^34^, the discharge of proteases (one category of hydrolases) should have theoretically stimulated invasion by degrading the extracellular matrix as is observed in hepatocellular carcinomas^34^. In melanoma cells, the loss of *IGF2R* expression also results in an increase in the secretion of proteins with an m6p tag, but hydrolases are activated at acidic pH, which can be found in the hepatocellular microenvironment but not in melanomas.

In order to confirm our finding in vivo, we invalidated *GNPTAB* in short-term culture WM1716 human melanoma. Those cell lines were subsequently engrafted into NSG mouse ears and the developement of metastasis was monitored. Our results indicated that while engraftment of WT WM1716 cells induced metastasis in the lung, the engineered cell line lost this ability. We could also observe that tumor growth at the injection site appeared lower with *GNPTAB* knock out cell line compared to WT when measured by bioluminescence intensity. However, when Ki67 index was measured, we did not observe significant differences between these two conditions indicating that cell proliferation is not dramatically affected. Moreover, we and others have demonstrated that melanomas with a high proliferative rate have lower invasive potential than slower ones and vice versa^2^. This indicates that even if in vivo the growth of invalidated cell line is potentially slightly affected, it does not interfere with the inhibition of invasion induced by impairing hydrolase transport.

Because lysosomes are key players in autophagy ^35^, we investigated if, in our conditions, this function was affected. When using siRNA mediated knock-down of *IGF2R* or *GNPTAB*, we did not observe the modulation of any autophagic marker. However, as expected, the genetic invalidation of *GNPTAB* blocked it. Those results indicated that although autophagy inhibition has been reported to prevent invasion^36^, short-term inhibition of hydrolase transport led to the same consequence independently.

In an effort to understand how lysosomal stress was induced by inhibiting hydrolase transport, we investigated the consequence of blocking this transport on lysosome biogenesis and observed an increase in those organelle number. Lysosome biogenesis is mainly regulated by TFEB, which translocates from the lysosome surface to the nucleus to stimulate the transcription of its targets in response to lysosomal stress^37^. However, the translocation of this transcription factor has been mainly studied in HeLa cells by overexpressing GFP-tagged TFEB^37^. The analysis of location of endogenous TFEB is to date a technical challenge as there is no satisfactory commercial antibody for immunostaining. In the melanocyte lineage, the question of localization of this transcription factor is an important issue as it shares common transcriptional targets with the other factors from the MiT family. In this group of transcription factors, MITF is cytoplasmic in all cell types except for melanocytes and melanomas^38^. This difference is explained by the fact that the melanocytic lineage expresses a shorter form of the protein lacking domain involved in its cytoplasmic retention. Therefore we cannot exclude that in melanoma a different isoform of TFEB could be expressed and differently regulated. This claim is partly supported by our data showing that lysosomal stress stimulates the expression of *TFEB*. Finally, we demonstrated that imparing hydrolase transport decreased melanoma invasiveness potential by upregulating *TFEB* expression. Those data were consistent with observations done on A375 melanoma cells as the level of expression of this gene directly affects their invasiveness potential^2^.

In conclusion, our results demonstrated that inducing a lysosomal stress by impairing hydrolase transport is a powerfull method to prevent melanoma invasion. This finding paves the road for the use of drugs that affect this transport to block melanoma tumor progression.

## Methods

### Cell culture

Wistar Melanoma 1716 were purchased from Rockland Immunochemicals. Wistar Melanoma 1745 short-term cultured cells were provided by Dr. Levi A. Garraway (Dana-Farber Cancer Institute, Boston, MA, USA). They were cultured in RPMI medium (Gibco) supplemented with 10% FBS (Gibco) and 1% penicillin-streptomycin antibiotics (Gibco). MDA-MB-231 breast cancer cells and neuroblastoma cells SK-N-SH and SK-N-AS were provided by Dr Joelle Wiels laboratory (Gustave Roussy, Villejuif, France). They were cultured in DMEM medium (Gibco) supplemented with 10% FBS and 1% penicillin-streptomycin antibiotics. All cell lines were maintained at 37 °C and 5% CO_2_.

### siRNA transfection

Small-interfering RNA against *IGF2R*, *GNPTAB* or *TFEB* (siGenome SMARTpool, Dharmacon) were transfected at 10 nM using lipidoid delivery agent C12-133-B as described by ^39^. All experiments performed with siRNA-depleted cells were assayed before 48 and 72 h after transfection to optimize gene inhibition efficiency.

### RNA purification and real-time reverse transcriptase-PCR

Total RNA was purified using TriZol reagent (Invitrogen) according to the manufacturer’s instructions. RNA was quantified by measuring optical density at 260 and 280 nm with Nanodrop device (ThermoFisher). RNA was subjected to cDNA synthesis using MultiScribe Reverse Transcriptase (Applied Biosystems) then amplified in SYBR reagent (Applied Biosystems) with StepOne Plus Real Time PCR (Applied Biosystems). Relative expression was normalized to *ACTB*. All primer sequences are shown in Supplementary Table 1.

### Western blotting

Melanoma cells were cultured in six-well dishes then transfected with indicated siRNA. 72 h after transfection, cells were lysed in buffer containing 50 mmol/L Tris-HCl (pH 7.4), 150 mmol/L NaCl, 1% Triton X-100, protease and phosphatase inhibitor cocktail (Thermo Fisher Scientific). Samples were dosed using Pierce BCA Protein Assay kit (ThermoFisher) and 30 µg of total extracts were resolved by 10% SDS-PAGE, transferred to nitrocellulose membranes, and then exposed to the appropriate antibodies: anti-βactin-HRP (Sigma-Aldrich A3854, diluted at 1: 5000), Vinculin (Cell Signaling Technology 13901 S, diluted at 1:1000), TFEB (Cell Signaling Technology 4240 S, diluted at 1:1000), LC3A/B (Cell Signaling Technology 4108, diluted at 1:1000), IGF2R (Cell Signaling Technology 14364 S, diluted at 1:1000), Cathepsin B (Cell Signaling Technology 31718, diluted at 1:1000), TFE3 (Cell Signaling Technology 14779 S, diluted at 1:1000), Phospho-pSMAD2 (Cell Signaling Technology 18338, diluted at 1:1000), SMAD2 (Cell Signaling Technology 5339, diluted at 1:1000), laminA/C (Cell Signaling Technology 2032, diluted at 1:1000), Phospho-4E-BP1 (Ser65) (Cell Signaling Technology 9451, diluted at 1:1000), 4E-BP1 (Cell Signaling Technology 9644, diluted at 1:1000).

Proteins were visualized with the ECL system (GE Healthcare) using horseradish peroxidase conjugated secondary antibodies.

### Invasion and migration assays

Cells were transfected with siRNA as described above. At 48 h after transfection, cells were serum-starved overnight. 5×10^4^ cells were added to invasion chambers, coated with reduced growth-factor Matrigel (Corning, 356230) for invasion assays. Cells were allowed to invade for 16 h toward media containing 10% fetal bovine serum. Cells remaining on the top side of the membrane were removed using a cotton swab. Invading cells present at the lower side of the membrane were fixed with cold methanol and stained with DAPI contained in Vectashield mounting medium (Vector Laboratories). Samples were analyzed in triplicate. All the surface of the membrane was photographed. The number of invaded cells was counted using CellSens imaging software (Olympus) and adjusted regarding proliferation rate of all seeded cells using crystal violet staining as viability assay. Plasmin at a concentration of 0.2–0.5 U/mL and TGFβ at a concentration of 0.1–0.5 ng/mL were added when indicated at cells seeding in Boyden chambers.

### Subcellular fractionation

Subcellular fractions were obtained using NE-PER Nuclear and Cytoplasmic Extraction kit purchased from Thermo Fisher according to manufacturer instructions.

### Autophagy stimulation

Autophagy conditions were applied to WM1716 by FBS starvation for 20 h and then cells were lysed to be analyzed for autophagic markers by immunoblotting LC3 proteins. Hydroxychloroquine (Sigma-Aldrich) at 50μM was used at as positive control of autophagy inhibition.

### Lysosome staining and confocal imaging

Cells plated on glass coverslips were incubated with LysoTracker Red DND-99 (0.5 μM; L7528, ThermoFisher) diluted in culture medium to stain the lysosome compartments for 1.5 h at 37 °C. Then they were fixed with 4% paraformaldehyde (ThermoFisher) for 20 min at room temperature (RT) and stained with DAPI diluted in 1% bovine serum albumin. Coverslips were mounted with Fluoromount medium (Clinisciences). They were imaged using Sp8 confocal microscope (Leica) with × 63 objective and LASX imaging software. Images were collected at 1024 × 1024 pixel resolution. Stained cells were optically sectioned along z axis. The total number of lysosomes were counted using a 3D reconstruction combining all z-stacks images with Volocity software (Volocity 3D Image Analysis Software, RRID:SCR_002668).

### CRISPR-Cas9 invalidation

CRISPR guides targeting *GNPTAB* were designed using crispr.mit.edu software from the Zhang lab^40^. Selected guide gtaaacaacgtcaatcggca, targeting GNPTAB downstream of its ATG was cloned into pL-CRISPR.SFFV.GFP (a kind gift from Benjamin Ebert; Addgene plasmid #57827). The generated vector was then transfected for 48 h into WM1716 short-term culture melanoma cells using Fugene HD (Promega) according to manufacturer’s specifications, before single cell sorting into 96 well dishes. Once the cultures were established, the clones were screened by sequencing of *GNPTAB*.

### Mannose-6-phosphate tagged protein purification and identification by LC-MS/MS

4 × 10^8^ cells per culture where flash frozen in liquid nitrogen before to be stored at -80 °C before to be processed by Peter Lobel laboratory at Rutgers University using the protocol described in. Briefly, cellular extracts were loaded on Affigel columns (Biorad) previously coupled with Soluble bovine cation-independent MPR (sCI-MPR). After immobilization of mannose-6-phosphate glycoproteins, the columns were washed prior elution with a solution containing 10 mM free mannose-6phosphate. The eluates were then subjected to mass spectrometry and analyzed using MS-Fit algorithm of the Protein Prospector suite.

### Cathepsin B activity assay

1 × 10^5^ cells were processed according to the manufacturer protocol (InnoZyme^TM^ Cathepsin B activity assay kit, Calbiochem).

### Mice experiment

NOD-SCID-IL2γ null (NSG) mice were purchased from Jackson Laboratory. Animal studies were performed with approval from the University of Tel Aviv Institutional Animal Care and Use Committee (M-11-053). For melanoma xenografts, 6×10^4^ cells from melanoma WM1716 expressing luciferase were injected with reduced growth-factor Matrigel (Corning, 356230) in ears of 6-week old NSG mice. Mice were monitored 7 weeks after melanoma injection for the development of tumors by measurements of tumor weight, tumor length (L), and width (W); tumor volume was calculated according to the formula (length x width^2^)/2. They were also tracked for bioluminescence emission using IVIS image system (Perkin Elmer, Santa Clara, CA, USA) after 2% isoflurane in oxygen incubation. Finally, they were all sacrificed, and their lungs were dissected to perform ex vivo bioluminescence analysis for metastasis detection. Images were analyzed with the Perkin Elmer software.

### Histology

Animals were euthanized by CO2 inhalation prior dissection. Tumor biopsies were kept in 10% buffered formalin until paraffin embedding and sectioning. Hematoxylin and eosin (H&E) and Ki67 (abcam, ab15580) staining was performed according to routine procedures.

### Statistics and reproducibility

All values for statistical significance represent the mean ± standard deviation (s.d.). The number of experimental repeats ‘n’ is stated throughout. Statistical analysis were performed using Graphpad Prism software (GraphPad Prism, RRID:SCR_002798). Statistical differences were calculated using ANOVA or t-tests when data were following normal distribution, otherwise Kruskal and Wallis tests were used. Differences were considered to be statistically significance for *p* < 0.05.

### Reporting summary

Further information on research design is available in the Nature Portfolio Reporting Summary linked to this article.

### Supplementary information

Supplemental information

Description of additional supplementary files

Supplementary Data

Reporting Summary

## Data Availability

The numerical source data can be found in Supplementary Data. All other data are available from the authors on reasonable request.
