# Supplementary material for: Impairing hydrolase transport machinery prevents human melanoma metastasis
Source: Commun Biol. 2024 May 15;7:574. doi: 10.1038/s42003-024-06261-y (PMC11096325; doi:10.1038/s42003-024-06261-y)
Supplement: Supplementary file 1 — Supplemental information [file 42003_2024_6261_MOESM1_ESM.pdf]

a.

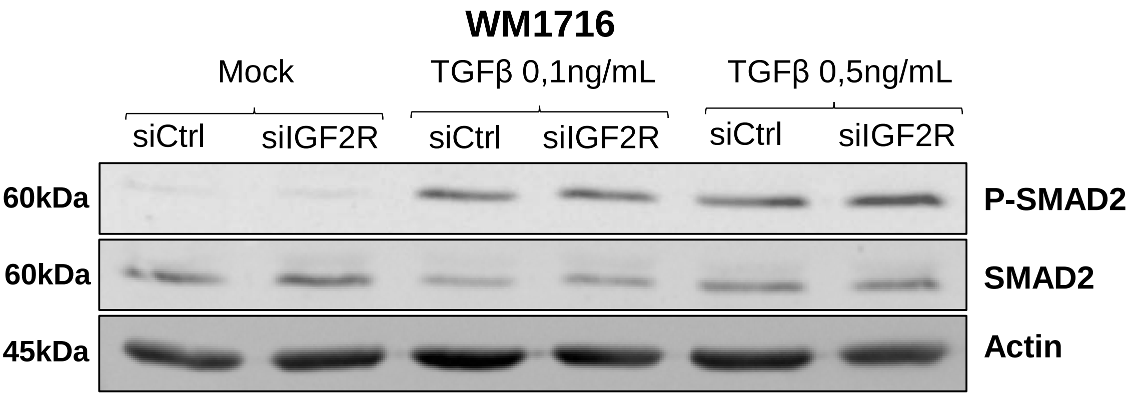

b.

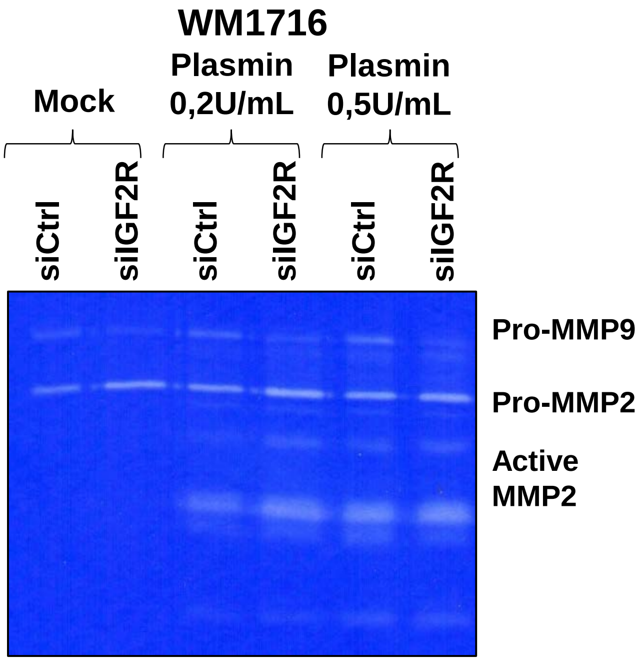

Supplementary Figure 1: Supplementation of WM1716-siIGF2R depleted cells with active TGFβ could promote SMAD2 phosphorylation and with active plasmin induced MMP2 cleavage. (a) Protein extracts from WM1716 siCtrl or siIGF2R treated with different concentrations of active TGFβ were analyzed by western blot for phosphorylated SMAD2. Actin served as a loading control. (b) Protein extracts from WM1716 siCtrl or siIGF2R treated with different concentrations of active plasmin were analyzed by gelatin zymography for MMP2 and MMP9 detection.

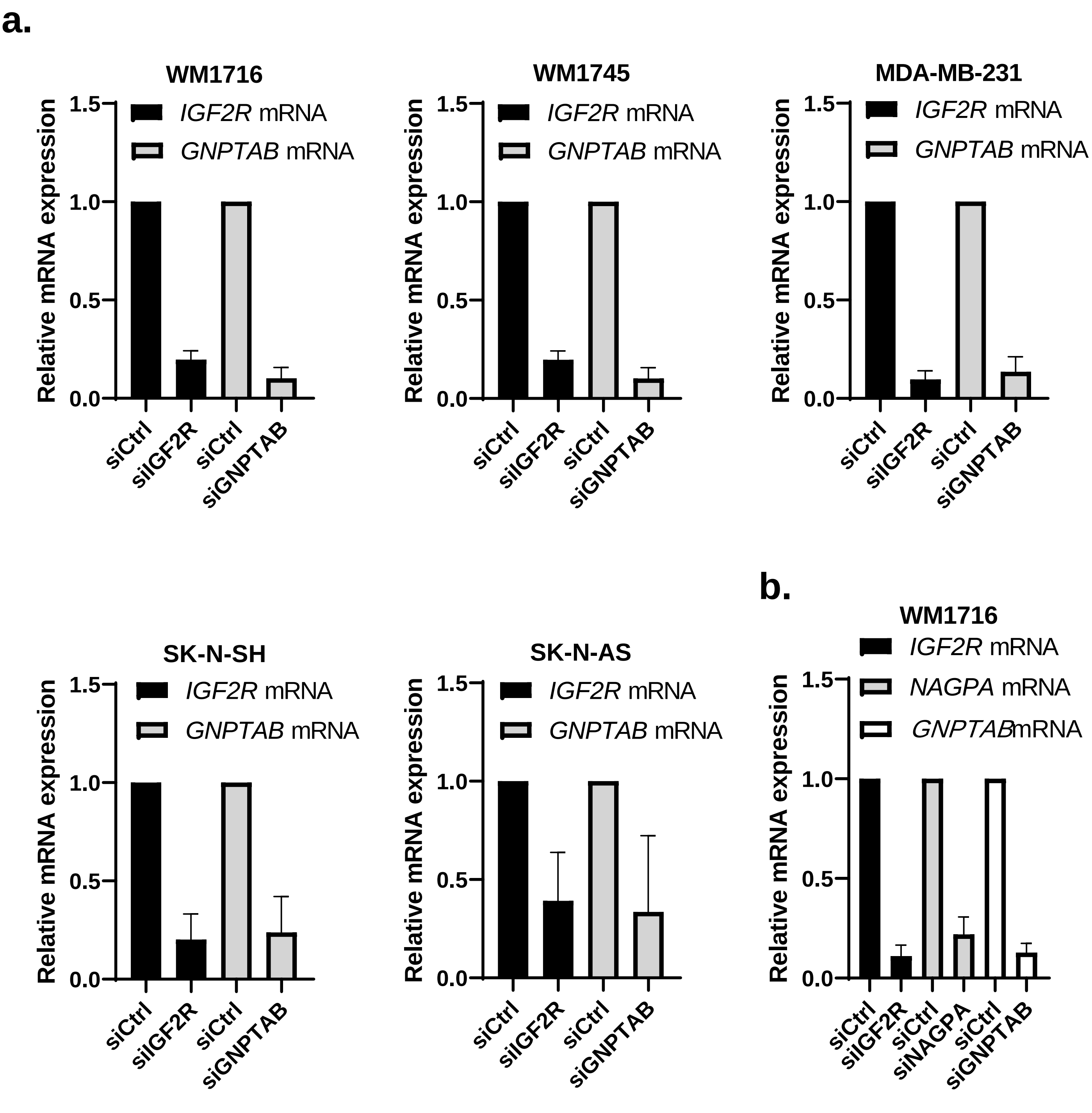

Supplementary Figure 2: Validation of *IGF2R*, *GNPTAB* and *NAGPA* depletion by siRNA. (a) Each indicated cell line were transfected with either siRNA specific for *IGF2*R or *GNPTAB* prior to be analysed by qRT-PCR. Each bar represent the relative expression of the mRNA targeted by the siRNA. The results are the average of at least three independent experiments. Error bars represent ± SD. (b) WM1716 cell line, transfected with indicated siRNA were analysed by qRT-PCR. Each bar represent the relative expression of the mRNA targeted by the siRNA. Error bars represent ± SD.

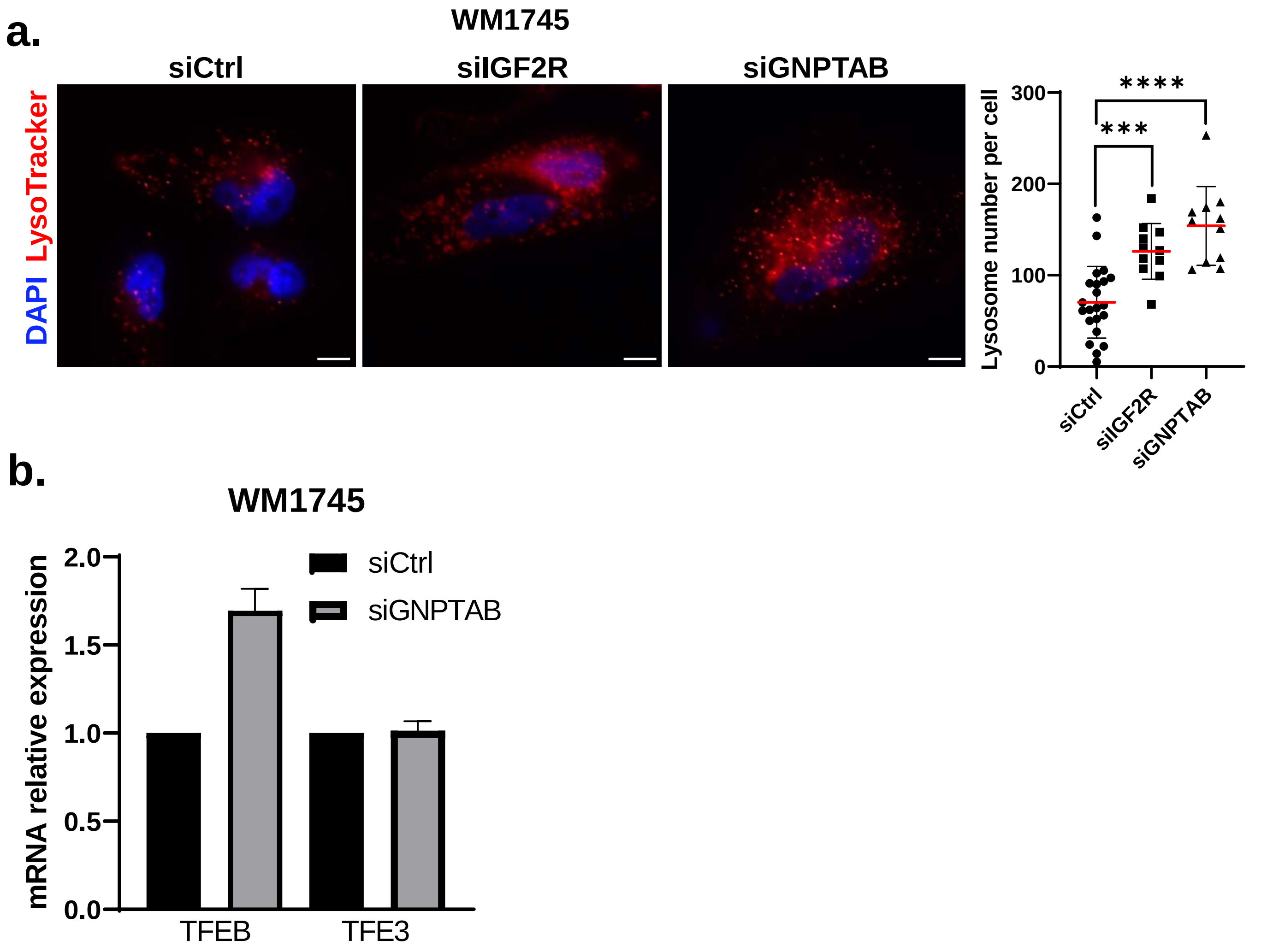

Supplementary Figure 3 : (a) Immunofluorescence confocal microscopy images of WM1745 cells transfected with indicated siRNA and stained with Red LysoTracker (red) and DAPI (blue). The quantifications of lysosomes numbers are shown on the right panel. Central bars indicate the average number of lysosomes per cell. One-way anova tests were used for statistical analysis. Error bars represent ± SD, ** indicates $p<0.01$, *** $p < 0.001$, **** indicates $p < 0.0001$. Scale bar = 10µm. (b) WM1745 cells were transfected with either control siRNA or specific for *GNPTAB* prior to be analysed by qRT-PCR. Each bar represent the relative expression of the mRNA targeted by the siRNA. The results are the average of at least three independent experiments. Error bars represent ± SD.

a.

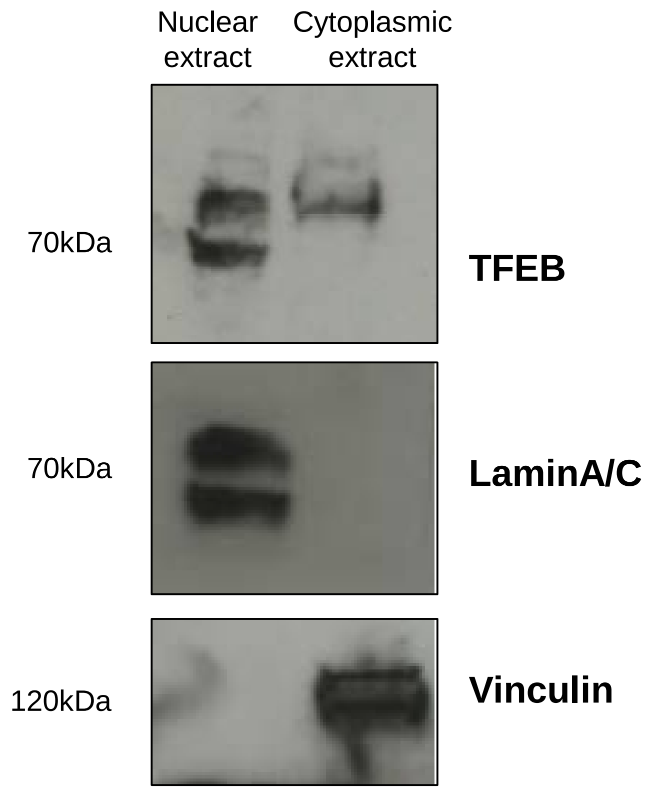

b.

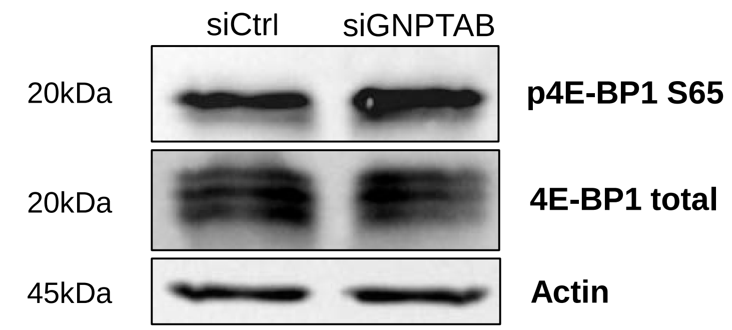

Supplementary Figure 4: TFEB is present inside the nucleus under phosphorylated and unphosphorylated forms in basal conditions in WM1716 cells. (a) WM1716 cells under basal conditions were lysed and fractioned using NE-PER fractionation kit (ThermoFisher). Cytoplasmic and nuclear fractions were analyzed by immunoblotting against TFEB. Lamin A/C served as a nuclear marker and vinculin served as a cytoplasmic marker. (b) Protein extract from WM1716 cells exposed to indicated siRNA were subjected to western blot analysis using antibody raised against total or S65 phosphorylated from of 4E-BP1.

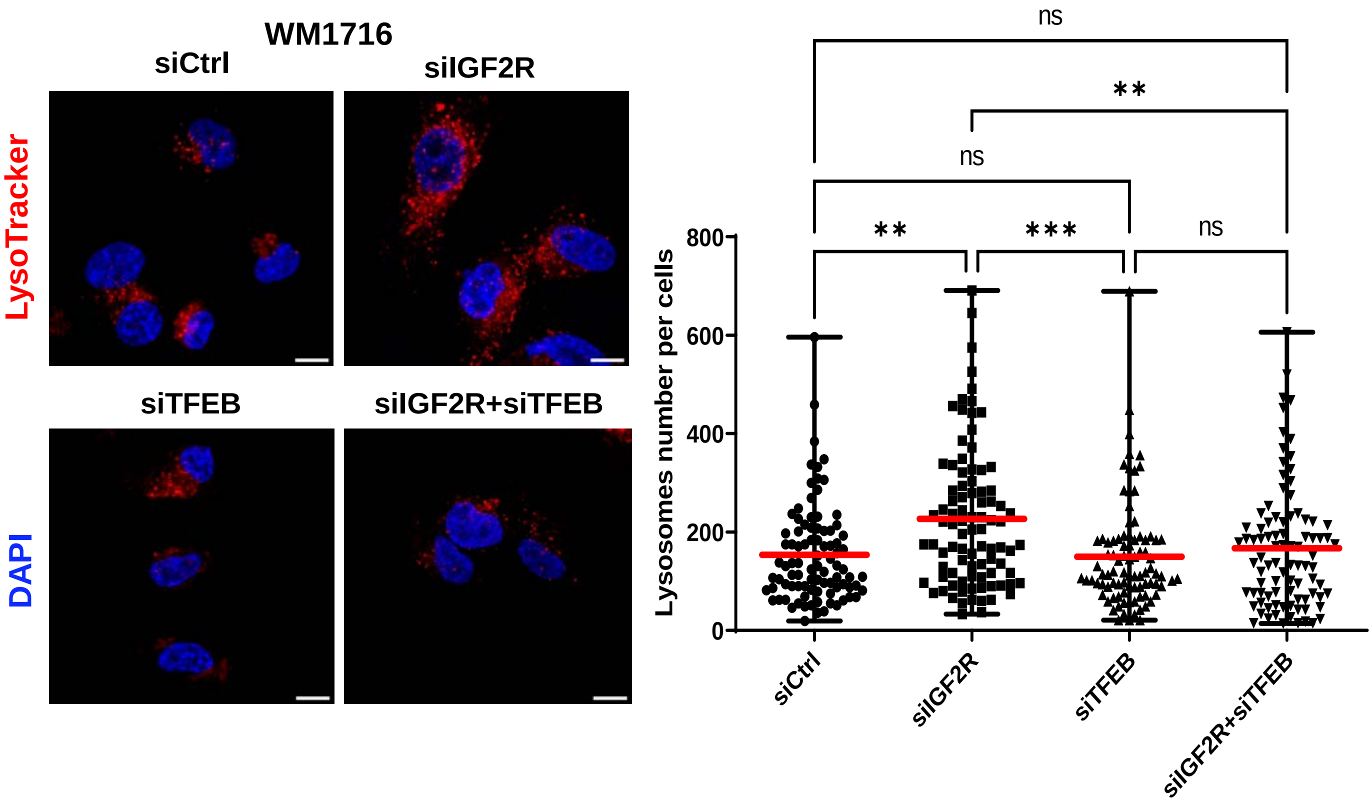

Supplementary Figure 5 : Modulation of *TFEB* expression regulates lysosome biogenesis of melanoma cells. WM1716 cells were transfected with indicated siRNA and stained with Red LysoTracker and DAPI (upper panel). The number of lysosomes per cell was quantified using Volocity software. Central bars indicate the average number of lysosomes per cell (lower panel). Kruskal and Wallis tests were used for statistical analysis. Error bars represent ± SD, ** indicates $p<0.01$, *** $p < 0.001$, **** indicates $p < 0.0001$. Scale bar = 10μm.

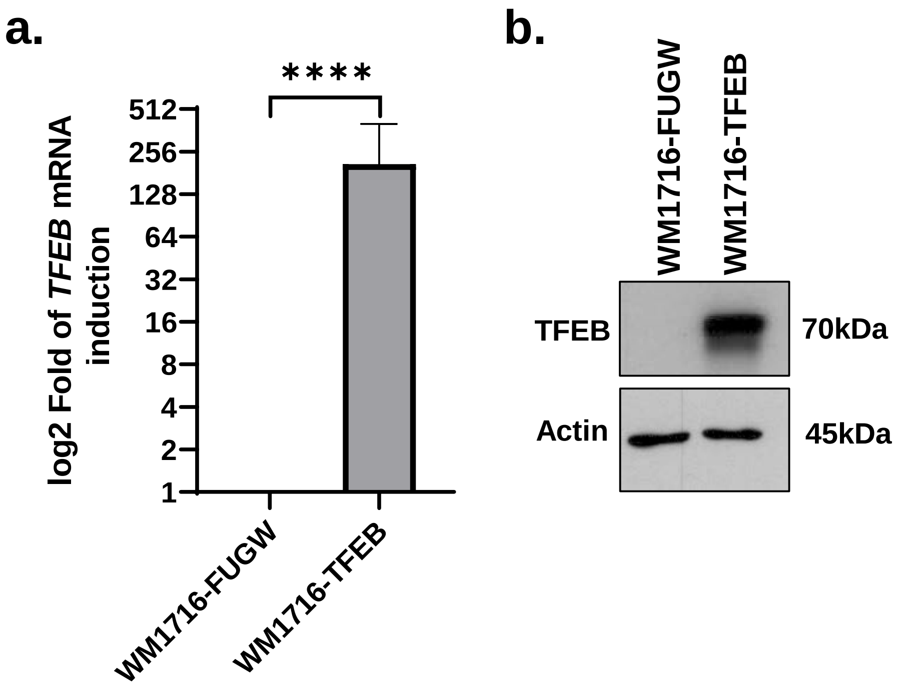

Supplementary Figure 6: Characterisation of WM1716 cell line ectopically expressing *TFEB* (a) *TFEB* mRNA expression comparison between WM1716 transduced with empty vector (FUGW) or with a lentivirus coding for TFEB (WM1716-TFEB). Results are shown as fold over control. Error bars represent ± SD. (b) Western blot analysis of protein extracts from WM1716-FUGW and WM1716-TFEB cell lines using TFEB and actin antibodies. (c) Number of WM1716-FUGW and WM1716-TFEB invasive cells assessed by matrigel coated transwell assay. Unpaired t-test was used for statistical analysis. Error bars represent ± SD.**** indicates $p < 0.0001$ (n=3 independent experiments).

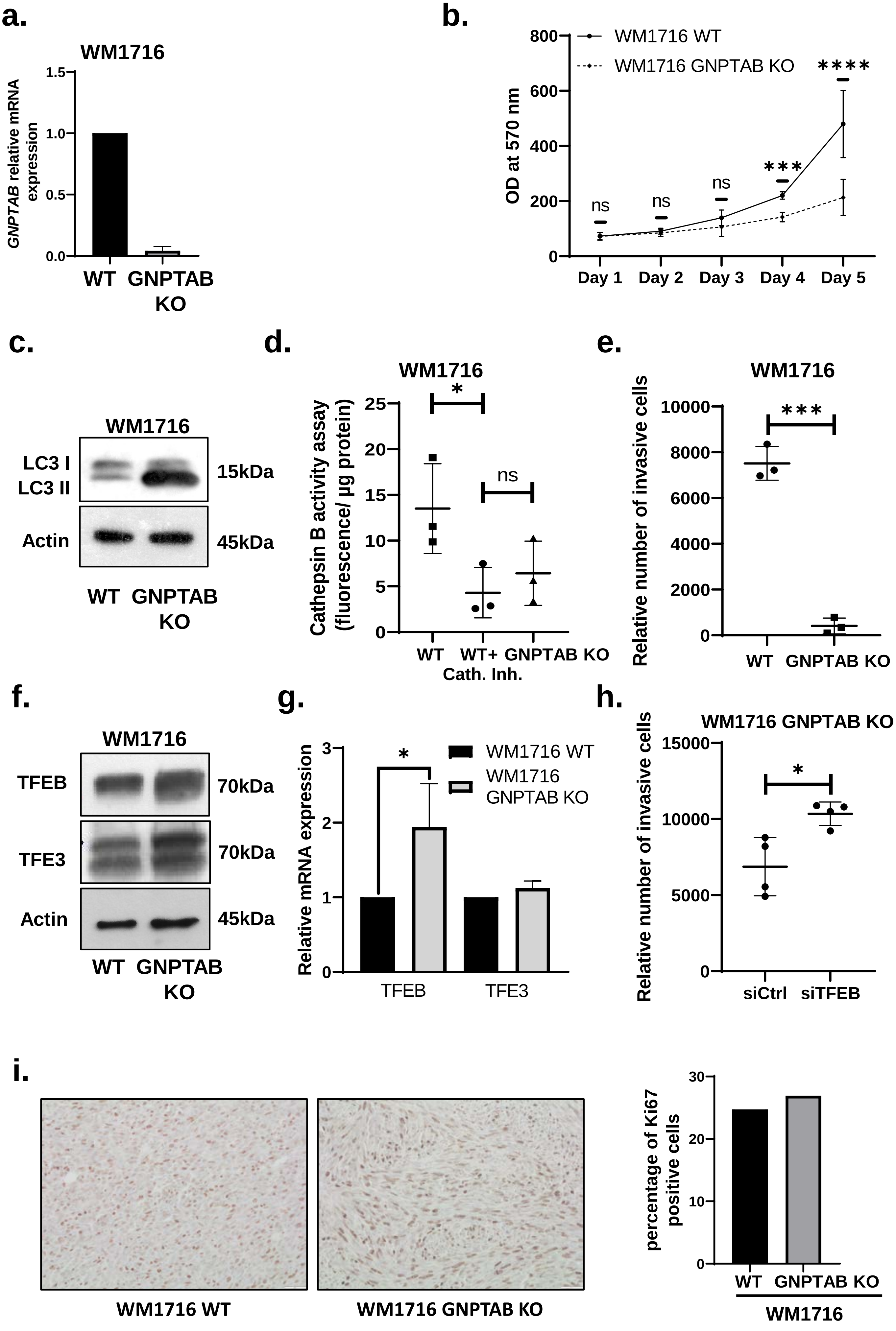

a.
WM1716
GNPTAB relative mRNA expression
1.5
1.0
0.5
0.0
WT
GNPTAB KO
b.
WM1716 WT
WM1716 GNPTAB KO
OD at 570 nm
800
600
400
200
0
ns
ns
ns
***
****
Day 1
Day 2
Day 3
Day 4
Day 5
c.
WM1716
LC3 I
LC3 II
15kDa
Actin
45kDa
WT
GNPTAB KO
d.
WM1716
Cathepsin B activity assay (fluorescence/ µg protein)
25
20
15
10
5
0
*
ns
WT
WT+ Cath. Inh.
GNPTAB KO
e.
WM1716
Relative number of invasive cells
10000
8000
6000
4000
2000
0
***
WT
GNPTAB KO
f.
WM1716
TFEB
70kDa
TFE3
70kDa
Actin
45kDa
WT
GNPTAB KO
g.
Relative mRNA expression
3
2
1
0
*
WM1716 WT
WM1716 GNPTAB KO
TFEB
TFE3
h.
WM1716 GNPTAB KO
Relative number of invasive cells
15000
10000
5000
0
*
siCtrl
siTFEB
i.
WM1716 WT
WM1716 GNPTAB KO
percentage of Ki67 positive cells
30
20
10
0
WT
GNPTAB KO
WM1716

Supplementary Figure 7: Characterisation of GNPTAB KO WM1716 cell line. (a) Total mRNA from WT and GNPTAB KO WM1716 cell lines were subjected to qRT-PCR using specific primer for *GNPTAB*. Each bar represents the relative expression of *GNPTAB*. Error bars represent ± SD. (b) Proliferation rates of WT and GNPTAB KO WM1716 cell lines were assessed by Crystal Violet staining and measurement of coloration at 570nm. Error bars represent ± SD, (n=3). (c) Western blot analysis of protein extracts from WT and GNPTAB KO cell lines using LC3 and actin antibodies. (d) Cathepsin B activity in WT WM1716 with or without Cathepsin B inhibitor (provided in the kit) and GNPTAB KO WM1716 cell lines. One-way anova was used for statistical analysis. Error bars represent ± SD (n=3). (e) Number of WT WM1716 or CRISPR GNPTAB WM1716 invasive cells assessed by matrigel coated transwell assay. Unpaired t-test was used for statistical analysis. Error bars represent ± SD (n=3). (f) Western blot analysis of protein extracts from WT and GNPTAB KO cell lines using TFEB, TFE3 and actin antibodies. (g) Total mRNA from WT and GNPTAB KO WM1716 cell lines were subjected to qRT-PCR using primers specific for TFEB or TFE3. (h) Relative number of invasive GNPTAB KO WM1716 melanoma cells measured by Matrigel coated transwell assays, after transfection with siCtrl or siTFEB (n=4). Unpaired t-test were used for statistical analysis. Error bars represent ± SD, ** indicates $p < 0.05$. (i) Ki67 staining by immunohistochemistry of indicated subcutaneous melanoma sections. Images were taken at 200X magnification. Scale bars = 50µM. The quantification of Ki67 cells is shown on the left panel bar graph. * indicates $p < 0.05$, **** indicates $p < 0.0001$.

HsRT-ACTINF	ATTGCCGACAGGATGCAGAA
HsRT-ACTINR	GCTGATCCACATCTGCTGGAA
HsRT-GNPTABF3	GTCACTCCAAAGGCCAGAA
HsRT-GNPTABR3	CTTGTCACAATAGCCATCCTTAATC
HsRT-LAMP1F	ACGTTACAGCGTCCAGCTCAT
HsRT-LAMP1R	TCTTTGGAGCTCGCATTGG
HsRT-IGF2RF	CACCAAGTAGGCACCACTA
HsRT-IGF2RR	GTGACCAGCAAGGCACAAA
HsRT-TFEBF	ACCTGTCCGAGACCTATGGG
HsRT-TFEBR	CGTCCAGACGCATAATGTTGTC
HsRT-TFE3F	TGCCTGTGTCAGGGAATCTG
HsRT-TFE3R	CGACGCTCAATTAGGTTGTGAT

Supplementary Table 1: Real Time PCR primers

SUPPLEMENTAL METHODS:

Gel zymography

Melanoma cells were cultured in 150 mm^2 dishes before to be washed twice with cold PBS prior to be incubated 15 min in 2 ml lysis buffer (Tris/HCl 0.025M pH 7.5, NaCl 0.1M, NP-40 1%, Aprotinin 10 µg/mL, Leupeptin, EDTA 0.004M). Then, protein concentrations were determined by BCA method (Pierce). Equal amount of protein was subjected to 0.1 % gelatin 10 % SDS-PAGE. After migration, the gel was renatured in a 2,5% Triton X-100 solution for 30 min prior to be washed twice with distillated water. Subsequently, the gel was incubated for 30 min shaking at room temperature in developing buffer (Tris/HCl 0.5M pH 7.8, NaCl 2M, CaCl2 0,05M and Brij-35 0,2%) and finally the gel was kept in fresh developing buffer for overnight at 37 °C. After 23 h incubation gels were stained with 0.1 % Coomassie blue for 1 h and destained in 5 % methanol and 10 % acetic acid. Zones of gelatinolytic activity were detected as clear bands against a blue background.

Figure1:Inhibitionof *IGF2R* expression prevents melanoma invasion

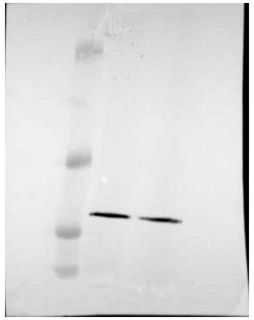

WM1716
vinculin

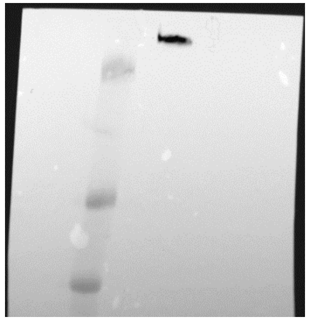

WM1716
IGF2R

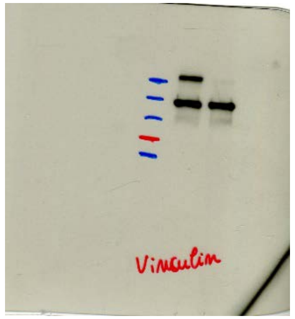

WM1745
vinculin

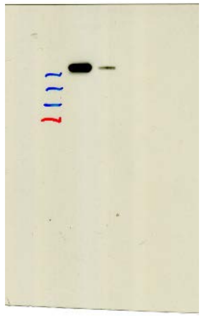

WM1745
IGF2R

Figure 4: *IGF2R*and *GNPTAB* inhibition increase lysosome number and *TFEB* and stimulate the expression of TFEB and *TFE3* expression.

a

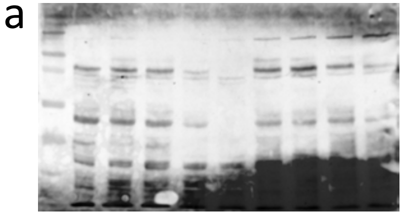

Anti-PL622 biotin / anti-streptavidine (1/2000^{e})

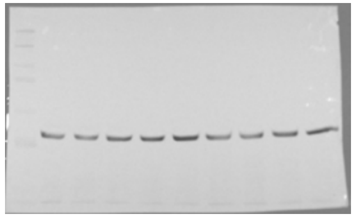

Actin

b

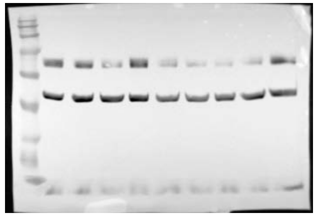

P62/ Actin

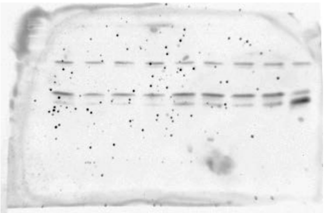

LC3 I/II

d

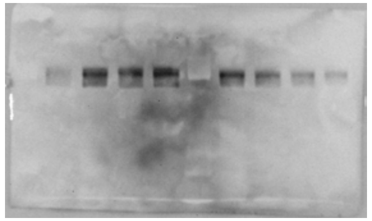

WM 1716
TFEB

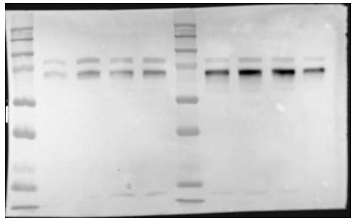

WM 1716
TFE3

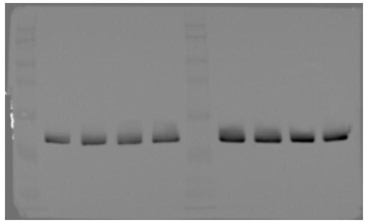

WM 1716
Actin

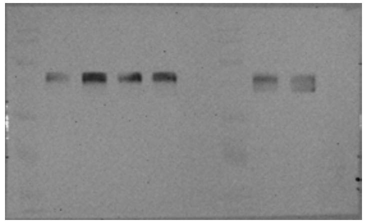

WM 1745
TFEB

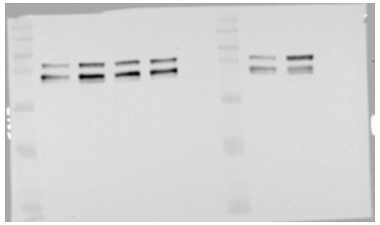

WM 1745
TFE3

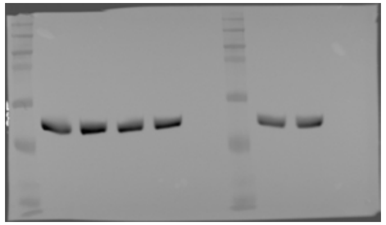

WM 1745
Actin

Supplementary Figure S1:

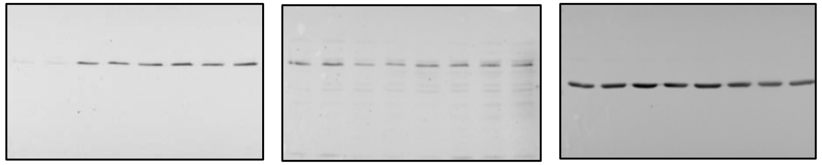

p-smad2 smad2 actin

Supplementary Figure S4:

a

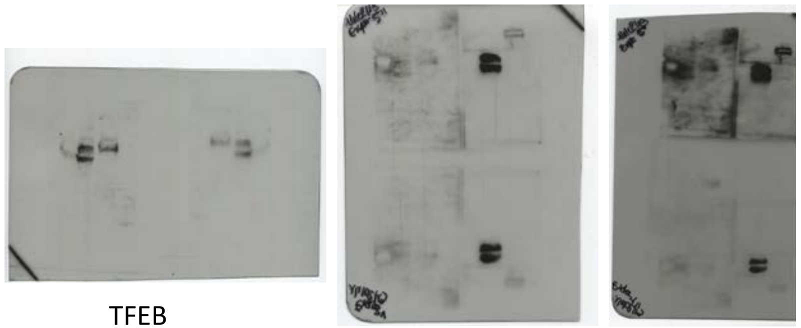

TFEB LaminA/C Vinculin

b

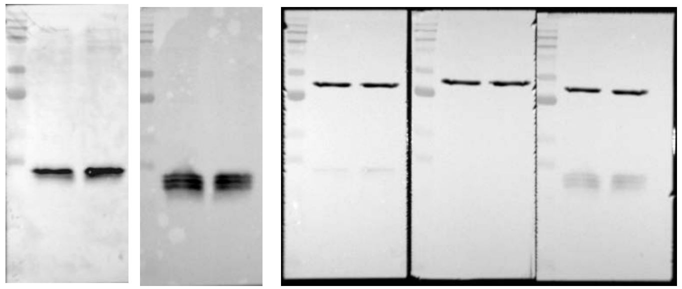

p4EBP1 S65 4EBP1 total actin

Supplementary Figure S6:

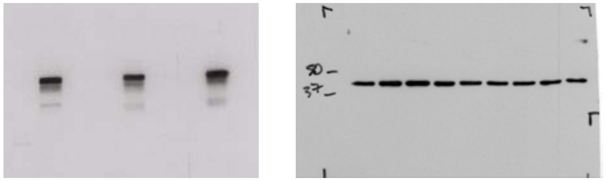

TFEB Actin

Supplementary Figure S7:

c

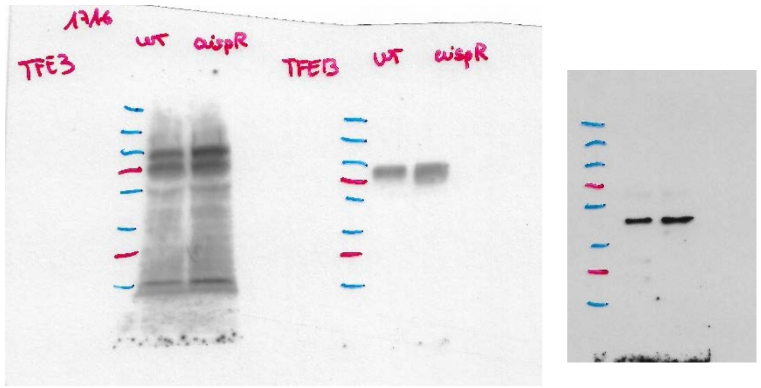

TFE3 TFEB Actin

f

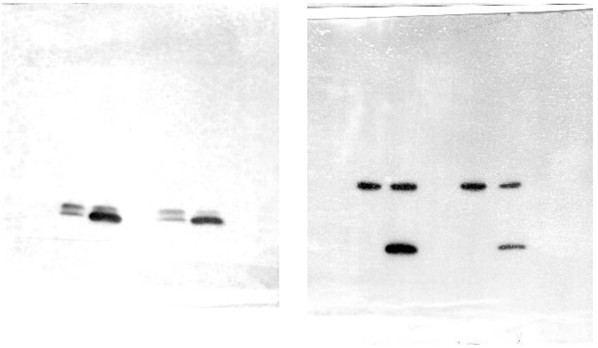

LC3 I/II Actin
