# Supplementary material for: Impairing hydrolase transport machinery prevents human melanoma metastasis
Source: Commun Biol. 2024 May 15;7:574. doi: 10.1038/s42003-024-06261-y (PMC11096325; doi:10.1038/s42003-024-06261-y)
Supplement: Supplementary file 2 — Description of additional supplementary files [file 42003_2024_6261_MOESM2_ESM.docx]

Description of Additional Supplementary Files

**File name:** Supplementary Data 1

**Description:** Numerical Source Data behind the graphs.
